# The caffeine dilemma: unraveling the intricate relationship between caffeine use disorder, caffeine withdrawal symptoms and mental well-being in adults

**DOI:** 10.1017/S1368980024000399

**Published:** 2024-02-02

**Authors:** Mahmut Bodur, Seda Kaya, Merve Ilhan-Esgin, Funda Pınar Çakiroğlu, Ayşe Özfer Özçelik

**Affiliations:** 1 Ankara University, Faculty of Health Sciences, Department of Nutrition and Dietetics, Ankara, Turkey; 2 Tokat Gaziosmanpaşa University, Faculty of Health Sciences, Department of Nutrition and Dietetics, Tokat, Turkey

**Keywords:** Anxiety, caffeine withdrawal, depression, mental health, stress

## Abstract

**Objective:**

This study aimed to explore the relationship between caffeine use disorder (CUD), caffeine withdrawal symptoms and the prevalence of depression, anxiety and stress (DASS) in adults.

**Design:**

The study utilised a cross-sectional design to assess the relationships between CUD, caffeine withdrawal symptoms and DASS.

**Setting:**

Participants’ CUD was evaluated through the Caffeine Use Disorder Questionnaire (CUDQ), while the Depression Anxiety Stress Scale-21 (DASS-21) measured DASS levels. Caffeine withdrawal symptoms and total caffeine intake were calculated based on self-reported consumption of caffeine-rich products.

**Participants:**

The study involved 618 participants with an average age of 27·8 (sd 7·8) years.

**Results:**

Participants consumed an average of 461·21 (sd 11·09) mg/d of caffeine, showing a positive correlation between CUD and total caffeine intake. The risk of CUD increased alongside levels of DASS. Individuals with caffeine withdrawal symptoms had higher CUDQ and DASS scores. A multiple linear regression model revealed significant associations between total caffeine intake (*P* < 0·001) and DASS-21 score (*P* < 0·001) with CUDQ score.

**Conclusions:**

The study concluded that caffeine, while recognised for its potential health benefits, also exhibits properties that may lead to addiction. The development of caffeine use disorder and cessation of caffeine intake can increase DASS levels in adults, indicating the need for awareness and appropriate interventions in public health nutrition.

Caffeine (1,3,7-trimethylxanthine) is a psychoactive substance found in a wide variety of beverages, such as coffee, tea, cola and energy drinks, as well as in some supplements, medications and foods like chocolate^(1,2)^. Caffeine is the most commonly consumed psychoactive substance in the world and unlike other psychoactive drugs is legal. Over the past decade, there has been a global increase in caffeine intake, with coffee being the most widely consumed caffeinated beverage worldwide^(3)^. In 2020/2021, the worldwide coffee consumption was reported to be approximately 166·63 million 60-kilogram bags^(4)^. This popularity of coffee can be attributed to its taste properties, stimulating effects and cultural significance in many countries.

Around 99 % of caffeine is absorbed from the gastrointestinal tract into the bloodstream, where it has various physiological effects, including cardiovascular and central nervous system stimulation^(5)^. Studies in adults have also reported some potential health benefits of moderate caffeine intake, such as analgesic effects and possible protective effects against certain medical conditions^(6,7)^. However, research also highlights that the increasing consumption of caffeine may be associated with psychological and physiological harm. Moreover, abuse and addiction to caffeine are becoming more common^(8)^. Potential harms from excessive caffeine consumption include acute toxicity, cardiovascular toxicity, bone and Ca effects, behavioural effects, impaired fetal development and subfertility^(9)^. Additionally, symptoms that do not meet clinical diagnostic criteria may occur more commonly, such as stress, headache, insomnia and feeling dependent^(10–12)^.

In 2013, caffeine use disorder was first included in the Diagnostic and Statistical Manual (DSM-5), and advanced study conditions confirm the potential clinical significance of a problematic syndrome resulting from heavy caffeine use. The primary purpose of including Caffeine Use Disorder in the DSM-5 is to encourage research that determines the reliability, validity and prevalence of caffeine use disorder based on the proposed diagnostic scheme^(13)^. Although it is known (accepted in the DSM-5) that caffeine can be an addictive substance with the potential to cause harm, there are significant research gaps. Prevalence estimates for caffeine use disorders are also rare. Current estimates are derived from a single review that includes only two very small studies from the general population (US and Italy)^(14)^ and a study with a recent, larger non-representative sample from Hungary^(15)^. Although there is a growing body of research on the medical harm from caffeine^(9,16)^, there is a lack of research in the literature that measures selected caffeine-related mental health outcomes and quality of life measures^(17)^.

Evidence for the relationship between caffeine intake and psychological disorders is limited^(7)^. Some previous studies have examined this relationship; however, their findings are inconsistent. Most studies have found an inverse relationship between caffeine intake and the risk of mental disorders^(18,19)^. Some have even reported that coffee or caffeine consumption increases the risk of depression, anxiety, sleep disorders and stress^(20,21)^. Several studies have shown that coffee, tea and chocolate have protective effects against depression^(22,23)^. Considering these studies, it is essential for public health to investigate the effect of consuming beverages containing high levels of caffeine, which are commonly consumed in relatively high amounts, on the psychological disorders experienced by individuals.

Most of the studies on the relationship between coffee and caffeine intake and mental health come from Western countries, and little information is available from developing countries^(22,24–26)^. Considering the nutritional transition in developing countries, the change in drinking habits from tea consumption to coffee consumption and the high prevalence of depression and anxiety in these countries, it is of great importance to evaluate the contribution of coffee and caffeine intake to these conditions. In addition, as far as we know, there are no studies evaluating the relationship between caffeine use disorder and symptoms of psychological disorders in the literature. Therefore, in this study, it is aimed to evaluate the relationship between caffeine use disorder and depression, anxiety and stress in the adult population.

## Material and methods

### Study sample and recruitment

This study consisted of 618 healthy individuals with a mean age of 27·8 (sd 7·8) years. Individuals with chronic and psychological disorders diagnosed by a doctor, requiring continuous medication use, were excluded. Additionally, individuals who regularly used dietary supplements (e.g. any mineral, vitamin) and/or drugs in the last 6 months were not included in the study. Those with caffeine restrictions or reductions due to medical conditions or treatment regimens were also excluded. No other inclusion or exclusion criteria were used.

The study was conducted between January and April 2021, and individuals were recruited through an online form developed by the researchers for this purpose. Before the study, all individuals were informed about the study, inclusion–exclusion criteria, and were given the choice to withdraw from the study if they did not wish to participate or met the exclusion criteria. The questionnaire included questions about the frequency of caffeinated food consumption, the Caffeine Use Disorder Questionnaire (CUDQ), Depression, Anxiety, and Stress Scale-21 (DASS-21), as well as caffeine withdrawal symptoms.

Individuals diagnosed with chronic diseases by a doctor (*n* 26) and individuals using continuous medication and/or dietary supplements (*n* 35) were excluded from the dataset before the analysis.

### Caffeine use disorder questionnaire and caffeine withdrawal symptoms

The Caffeine Use Disorder Scale was developed by Agoston et al. (2018)^(15)^. The scale, which consists of ten items, is of the four-point Likert type. The statements in the scale used to evaluate the last 12 months are scored as follows: ‘never’ receives one points, ‘sometimes’ receives two points, ‘often’ receives three points and ‘very often’ receives four points. A higher total score indicates a higher risk of caffeine disorder. The Turkish validity and reliability study of this scale was conducted by Kaya et al. (2023)^(27)^. In this study, Cronbach’s *α* coefficient of 0·870.

For assessing caffeine withdrawal symptoms (headache, fatigue or drowsiness, depressed mood or irritability, difficulty concentrating, flu-like symptoms and inconvenience or suffering in daily life), seven dichotomous items were used to determine whether the individuals experienced these symptoms after not consuming caffeine for 24 h or more during the past year^(28)^.

### Dietary caffeine intake

In order to determine the average amount of caffeine consumption by individuals in the last month, researchers created a form to record the frequency of caffeine-rich food consumption based on previous studies^(29)^ (see online supplementary material, Supplemental Table S1). Caffeinated foods were classified into three different categories: caffeinated beverages, foods containing cocoa and chocolate varieties. These categories were established considering the common consumption patterns of these products in Turkish society and a previous study conducted in Türkiye. The four groups of food and drinks included tea-based beverages (black tea, green tea), coffee-based beverages (Turkish coffee, filter coffee and instant coffee), other beverages (coke and energy drinks) and chocolate varieties (dark chocolate, milk chocolate, white chocolate, dragee and waffles).

To determine the consumption frequency, the form provided options such as ‘never,’ ‘every meal,’ ‘every day,’ ‘5–6 times a week,’ ‘3–4 times a week,’ ‘1–2 times a week,’ ‘once every 15 d,’ ‘once a month’ and ‘no consumption.’ Additionally, pictures of glasses, mugs and visuals were included to assist individuals in accurately and easily determining the amount consumed for various foods and beverages^(30)^. The caffeine content in caffeinated foods and beverages was obtained from the US Department of Agriculture and previous studies^(31)^.

### Depression, Anxiety, and Stress Scale-21 questionnaire

In this study, the DASS-21 scale was used to evaluate the individuals’ levels of depression, anxiety and stress. The DASS-21 is a four-point Likert-type scale that measures sub-dimensions of depression, anxiety and stress, with seven items for each sub-dimension^(32)^. Responses are scored as follows: ‘Always’ receives three points, ‘Quite often’ receives two points, ‘Sometimes’ receives one point and ‘Never’ receives zero points. The Turkish validity and reliability study of the DASS-21 scale was conducted by Sarıçam in 2018^(33)^. In this study, Cronbach’s *α* values for depression, anxiety and stress sub-dimensions are 0·84, 0·90 and 0·84, respectively. Higher scores in each subgroup of the scale indicate higher levels of depression, anxiety and stress.

### Statistical analyses

Statistical analyses were conducted using IBM SPSS 21 Statistics. Descriptive analysis included the use of mean ± sd for normally distributed variables and number (percentage) for categorised variables. The normality of the variables was assessed through visual methods (histogram and probability graphs) and analytical methods (Kolmogorov–Smirnov/Shapiro–Wilk test).

Anxiety, depression, stress and caffeine use disorder scale scores of the individuals were compared based on the presence of caffeine withdrawal symptoms. The Pearson *χ* ^2^ test was used to compare the significance of the difference between the two percentages. Pearson correlation was performed to investigate the relationships between key study variables, including CUDQ total scores, total caffeine intake and subgroups, as well as DASS-21 total and subscores.

The total score of the CUDQ was considered the dependent variable, and a multiple linear regression model was used to identify influencing factors. Model 1 showed the direct relationship, while Model 2 was adjusted for age and gender. In model 3, these variables were additionally adjusted for smoking status and marital status.

## Results

The demographic characteristics and scale scores of the individuals are shown in Table 1. According to the table, 81·2 % of the individuals are female, 70·7 % are single and 81·1 % are non-smokers. The mean BMI of individuals was 22·8 (sd 3·7) kg/m^2^, and 65·2 % of them had normal BMIs (18·5–25·0 kg/m^2^). The mean caffeine intake of the individuals was 461·21 (sd 11·09) mg/d, with the most significant contribution coming from tea. The mean CUDQ scores of the individuals were 16·3 (sd 5·5) points. When examining DASS-21 scores, the mean depression score was 5·3 (sd 0·2), the mean anxiety score was 4·6 (sd 0·2) and the mean stress score was 6·3 (sd 0·2). The total DASS-21 score of the individuals was 16·3 (sd 0·5) points. Considering the symptoms of caffeine withdrawal, 35·9 % of individuals experienced headaches, 35·4 % reported fatigue or drowsiness, 24·8 % had depressed mood or irritability and 30·3 % had difficulty concentrating. Additionally, 12·9 % experienced flu-like symptoms, and 10·8 % stated that they significantly suffered. The CUDQ score was predicted by anxiety, coffee and chocolate consumption, indicating their essential roles in estimating CUDQ scores. On the other hand, after adjusting for certain demographic and lifestyle factors, depression and stress scores may not be significant predictors (see online supplementary material, Supplemental Table S2, data is not shown)

**Table 1:** The demographic and key study variables of the participants

	*n*	%	min–max
Sex			
Female	502	81·2	
Male	116	18·8	
Age			
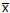	27·8		20·0–57·0
sd	7·8		
Marital status			
Single	437	70·7	
Married	181	29·3	
Smoking status			
No	501	81·1	
Yes	117	18·9	
BMI (kg/m^2^)			
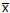	22·8		15·4–38·7
sd	3·7		
BMI categories			
Underweight (≤ 18·5 kg/m^2^)	60	9·7	
Normal (18·5–24·9 kg/m^2^)	403	65·2	
Overweight or obese (≥ 25·0 kg/m^2^)	155	25·1	
	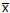	sd	
Caffeine intake (mg/d)	461·21	11·09	16·00–2089·56
Tea consumption	260·42	8·36	0·00–1164·00
Coffee consumption	21·90	1·62	0·00–391·00
Coke and energy drink consumption	21·90	1·62	0·00–391·00
Chocolate consumption	38·50	2·08	0·00–298·05
CUDQ total score	16·3	5·5	10·0–38·0
DASS-21 scores			
Depression scores	5·3	0·2	0–21·0
Anxiety scores	4·6	0·2	0–21·0
Stress scores	6·3	0·2	0·0–21·0
DASS-21 total score	16·3	0·5	0·0–63·0
Caffeine withdrawal symptoms			
Headache	222	35·9	
Fatique or drowsiness	219	35·4	
Depressed mood or irritability	153	24·8	
Difficulty concentrating	187	30·3	
Flu-like symptoms	80	12·9	
Significant suffering	67	10·8	

**Fig. 1:**
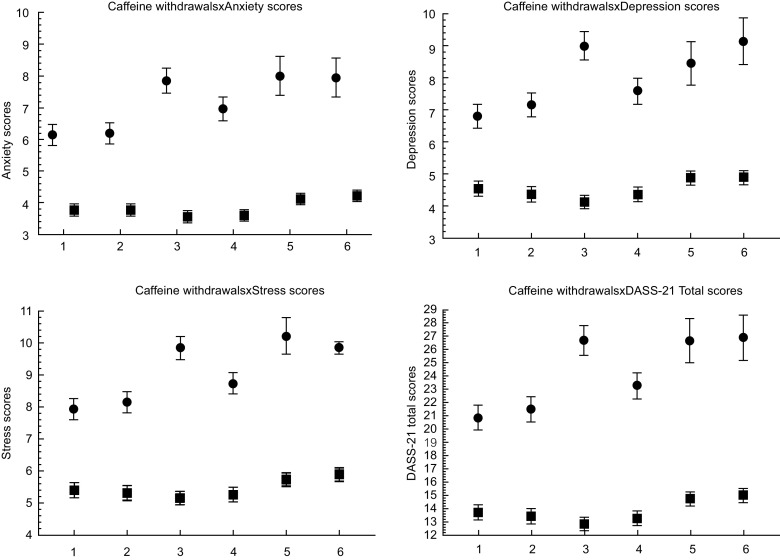
The relationship between caffeine withdrawal symptoms and CUDQ total scores. Caffeine withdrawal symptoms were dichotomous (yes or no), ● : participants with caffeine withdrawal symptoms (‘Yes’), ■ : participants without caffeine withdrawal symptoms (‘No’). Independent sample *t* test was used. CUDQ = caffeine use disorder questionnaire, caffeine withdrawals symptoms were questioned for last 1 month

Figure 1 and online supplementary material, Supplemental Table S3, show the DASS-21 subgroup and total scores based on the presence of caffeine withdrawal symptoms. Individuals experiencing symptoms related to caffeine withdrawal have higher depression, anxiety and stress scores compared with individuals who do not experience symptoms. Similarly, individuals with caffeine withdrawal had higher total DASS-21 scores. All pairwise comparisons were found to be statistically significant (*P* < 0·001).

**Fig. 2:**
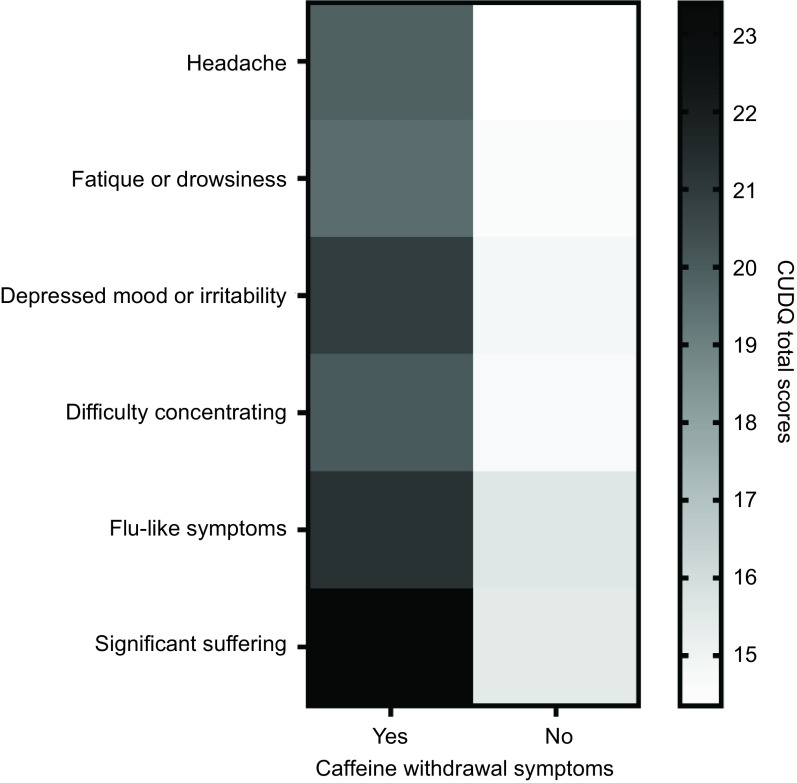
The correlation of caffeine withdrawal symptoms with CUDQ scores. CUDQ = caffeine use disorder questionnaire, caffeine withdrawal symptoms were dichotomous (yes or no), displays the distribution of dichotomous responses to caffeine withdrawal symptoms (Yes/No) in relation to CUDQ scores, using a colour scale. Darker shades represent a higher frequency of ‘Yes’ responses, while lighter shades indicate more ‘No’ responses. Independent sample *t* test was used

According to Fig. 2 and online supplementary material, Supplemental Table S4, the average CUDQ scores of individuals experiencing symptoms related to caffeine withdrawal were found to be higher. All pairwise comparisons were found to be statistically significant (*P* < 0·001).

Table 2 shows the multiple linear regression model indicating the relationship between CUDQ scores, DASS-21 scores and total caffeine intake. DASS-21 scores and total caffeine intake were found to be associated with CUDQ scores. This relationship remained significant even after adjusting for gender and age, as observed in model 3. Similarly, the significance persisted when adjusted for smoking status and marital status.

**Table 2:** Relationship between CUDQ as a dependent variable and DASS-21 total scores and total caffeine intake

	DASS-21 total scores			Total caffeine intakes		
	*β*	95 % CI	*P*	*β*	95 % CI	*P*
Model 1	0·176	0·147, 0·204	< 0·001^*^	0·008	0·005, 0·011	< 0·001^*^
Model 2	0·177	0·148, 0·205	< 0·001^*^	0·006	0·004, 0·009	< 0·001^*^
Model 3	0·176	0·148, 0·205	< 0·001^*^	0·005	0·004, 0·006	< 0·001^*^

CUDQ, caffeine use disorder questionnaire.

* *P* < 0·0001.

Model 1. Crude model.

Model 2. Including model 1 and adjusted for age and sex.

Model 3. Including model 2 and adjusted for marital status and smoking status.

## Discussion

Caffeine is frequently consumed in Türkiye, as in many other countries^(34)^. Despite the known benefits of caffeine, it has the potential to cause addiction^(8)^. The Türkiye Dietary Guideline recommends that caffeine intake should not exceed 400 mg/d^(35)^. However, in this study, the average caffeine intake of individuals was high (> 400 mg/d), associated with an increased risk of caffeine use disorder. A large-scale cross-sectional study conducted in New Zealand reported that individuals with higher caffeine intake were more likely to have a caffeine use disorder. Even moderate caffeine intake (200–400 mg/d) increased the odds of caffeine use disorder compared with low caffeine intake (< 200 mg/d)^(36)^. Sweeney et al. also found that as caffeine intake increased, the criteria for caffeine use disorder were met more frequently. Feeling guilty or bad about caffeine use was associated with caffeine use disorder. Similar to the present study, depression, anxiety and stress were also reported to be more common in people with caffeine use disorder^(37)^.

The mechanism underlying caffeine use disorder and its psychological effects is not fully understood. However, it is known that caffeine binds to adenosine receptors by competing with endogenous adenosine and reduces drowsiness^(5)^. In individuals who regularly consume caffeine, the number of adenosine receptors in the central nervous system increases, and individuals become more sensitive to the normal physiological effects of adenosine^(38)^. While caffeine may have a protective effect against depression up to a certain dose^(39)^, excessive caffeine intake can exacerbate depression and induce anxiety^(40)^. Caffeine primarily acts on two of the four adenosine receptors, A1 and A2a receptors^(41)^, which have been implicated in anxiogenic behaviours in animal studies^(42,43)^. It is also suggested that caffeine may influence anxiety by increasing the activity of the hypothalamic–pituitary–adrenal axis^(44)^.

Caffeine is regularly consumed in many societies and is used to provide alertness and reduce drowsiness^(26,45)^. However, caffeine can be addictive, and withdrawal symptoms may occur when caffeine intake is stopped. In the present study, it was observed that the CUDQ scores of individuals with caffeine withdrawal symptoms, one of the criteria proposed for caffeine use disorder, were higher, as expected. Approximately one-third of the individuals experienced headache, fatigue/drowsiness and impaired concentration as caffeine withdrawal symptoms. Individuals with withdrawal symptoms had higher depression, anxiety and stress scores. Magdy et al. also reported that on the first day of Ramadan fasting, 55 % of people reported headaches due to caffeine withdrawal, accompanied by other symptoms such as depressed mood, impaired attention and drowsiness^(46)^. In a study suggesting that thoughts are effective on addictions, Mills et al. showed that decaffeinated coffee, which is believed to be caffeinated, is effective in reducing withdrawal symptoms^(47)^. Similarly, in another study, a nocebo effect was observed in caffeine withdrawal symptoms when individuals were aware of dose reductions^(48)^. This may be beneficial in reducing withdrawal symptoms in individuals with caffeine use disorder.

### Strengths and limitations

One of the strengths of the study is that, to the best of our knowledge, it is the first study to evaluate caffeine use disorder in the Turkish population. In this study, with a relatively large sample size, caffeine use disorder was assessed using the CUDQ, a scale developed according to DSM-5 criteria that can assess the full spectrum of caffeine use disorder.

In addition to its strengths, this study also has some limitations. One limitation is that women were overrepresented in this study. Additionally, the data were collected online, and the assessment of caffeine use disorder, depression, anxiety and stress status was based on self-report data. Furthermore, it should be noted that the caffeine content of beverages such as tea and coffee can be influenced by brewing time and method, which may introduce variability in our assessment. In further studies, evaluating depression, anxiety and stress levels by an expert may provide a more precise determination of the psychological effects of caffeine use disorder.

### Conclusion

This study found that caffeine intake increased as the risk of caffeine use disorder increased. The analyses also showed that caffeine use disorder was associated with depression, anxiety and stress. Depression, anxiety and stress levels were found to be higher in individuals with caffeine withdrawal symptoms. The addictive nature of caffeine and withdrawal symptoms should be taken into consideration, especially in caffeine-sensitive populations and those with high caffeine intake. Awareness should be raised about the symptoms that can be experienced even if the clinical diagnostic criteria are not met.

Caffeine use disorder, which requires further research according to DSM-5, is a new area of research, and the literature in this area is quite limited. Research on caffeine use disorder tends to be cross-sectional and has some limitations, as in this study. Therefore, longitudinal studies should be conducted to clearly define caffeine use disorder and to clarify the diagnostic criteria.

## Supporting information

Bodur et al. supplementary material Bodur et al. supplementary material

## Supplementary material

For supplementary material accompanying this paper visit https://doi.org/10.1017/S1368980024000399
