# Supplementary material for: The caffeine dilemma: unraveling the intricate relationship between caffeine use disorder, caffeine withdrawal symptoms and mental well-being in adults
Source: Public Health Nutr. 2024 Feb 2;27(1):e57. doi: 10.1017/S1368980024000399 (PMC10882539; doi:10.1017/S1368980024000399)
Supplement: Bodur et al. supplementary material [file S1368980024000399sup001.docx]

**The Caffeine Dilemma: Unraveling the Intricate Relationship Between Caffeine Use Disorder, Caffeine Withdrawal Symptoms, and Mental Wellbeing in Adults**

**Supplementary tables**

| Food and Drinks | Caffeine content (mg / 100 g) |
| --- | --- |
| Caffeinated Drinks |  |
| Black tea (brewed) | 20 |
| Black tea (bag) | 24 |
| Green tea (brewed) | 18 |
| Green tea (bag) | 11 |
| Turkish coffee | 60 |
| Nescafe (classic) | 44 |
| Nescafe (gold) | 26 |
| Nescafe (decaffeinated) | 2 |
| Cappuccino | 11 |
| Nescafe 3 in 1 | 22 |
| Nescafe 2 in 1 | 23 |
| Hot chocolate | 2 |
| Chocolate milkshake | 3 |
| Chocolate milk | 2 |
| Cola | 12 |
| Diet cola | 16 |
| Energy drinks | 30 |
| Ice tea | 3 |
| Chocolate and foods with chocolate |  |
| Milk chocolate | 24 |
| Bitter chocolate | 68 |
| White chocolate | 0 |
| Chocolate waffles | 14 |
| Chocolate sticks | 3 |
| Chocolate dragee | 22 |
| Chocolate biscuits | 3 |
| Chocolate cake | 2 |
| Chocolate cookie | 2 |
| Chocolate ice cream | 2 |
| Chocolate pudding | 2 |
| Foods with cocoa |  |
| Cocoa cake | 5 |
| Cocoa cookies | 3 |
| Cocoa biscuits | 3 |
| Cocoa breakfast cereals | 3 |
| Cocoa pudding | 2 |

**Table S1.** Caffeine contents of food and drink

|  | **Model 1** | | **Model 2** | | **Model 3** | |
| --- | --- | --- | --- | --- | --- | --- |
|  | **β (95% CI)** | **p** | **β (95% CI)** | **p** | **β (95% CI)** | **p** |
| Anxiety scores | .300 (.177, .460) | <0.001** | .328 (.187, .468) | <0.001** | .340 (.159, .440) | <0.001** |
| Depression scores | .069 (-.063, .201) | 0.304 | .088 (-.044, .220) | 0.191 | .109 (-.023, .241) | 0.105 |
| Stress scores | .154 (.011, .297) | 0.034* | .131 (.012, .274) | 0.048* | .132 (-.010, .274) | 0.068 |
| Tea consumption | .001 (-.003, .004) | 0.641 | .001 (-.003, .004) | 0.717 | .001 (-.003, .004) | 0.725 |
| Coffee consumption | .006 (.004, .008) | <0.001** | .006 (.004, .008) | <0.001** | .006 (.004, .008) | <0.001** |
| Coke and energy drink consumption | .002 (-.008, .011) | 0.747 | .003 (-.007, .013) | 0.534 | .001 (-.011, .009) | 0.810 |
| Chocolate consumption | .009 (.002, .017) | 0.013* | .010 (.003, .017) | 0.008* | .010 (.003, .017) | 0.008* |

**Table S2.** Relationship between predictor of CUDQ as a dependent variable and DASS-21 subtotal scores and dietary caffeine sources

Model 1. Crude model

Model 2. Including model 1 and adjusted for age and sex

Model 3. Including model 2 and adjusted for marital status and smoking status

*p<0.05, **p<0.0001

| **Depression Scores/Caffeine Withdrawal symptoms** | **YES** | | **NO** | |  |
| --- | --- | --- | --- | --- | --- |
|  | **n** | **x̄ ± SD** | **n** | **x̄ ± SD** | **p** |
| Headache | 222 | 6.8±0.4 | 396 | 4.5±0.2 | <0.001** |
| Fatique or drowsiness | 219 | 7.2±0.4 | 399 | 4.4±0.2 | <0.001** |
| Depressed mood or irritability | 153 | 9.0±0.4 | 465 | 4.1±0.2 | <0.001** |
| Difficulty concentrating | 187 | 7.6±0.4 | 431 | 4.4±0.2 | <0.001** |
| Flu-like symptoms | 80 | 8.5±0.7 | 538 | 4.9±0.2 | <0.001** |
| Significant suffering | 67 | 9.1±0.7 | 551 | 4.9±0.2 | <0.001** |
| **Depression Scores/Caffeine Withdrawal symptoms** |  | **YES** |  | **NO** |  |
|  | **n** | **x̄ ± SD** | **n** | **x̄ ± SD** | **p** |
| Headache | 222 | 6.1±0.3 | 396 | 3.8±0.2 | <0.001** |
| Fatique or drowsiness | 219 | 6.2±0.3 | 399 | 3.8±0.2 | <0.001** |
| Depressed mood or irritability | 153 | 7.9±0.4 | 465 | 3.5±0.3 | <0.001** |
| Difficulty concentrating | 187 | 7.0±0.4 | 431 | 3.6±0.1 | <0.001** |
| Flu-like symptoms | 80 | 8.0±0.6 | 538 | 4.1±0.2 | <0.001** |
| Significant suffering | 67 | 7.9±0.6 | 551 | 4.2±0.2 | <0.001** |
| **Depression Scores/Caffeine Withdrawal symptoms** |  | **YES** |  | **NO** |  |
|  | **n** | **x̄ ± SD** | **n** | **x̄ ± SD** | **p** |
| Headache | 222 | 7.9±0.3 | 396 | 5.4±0.2 | <0.001** |
| Fatique or drowsiness | 219 | 8.2±5.3 | 399 | 5.3±0.2 | <0.001** |
| Depressed mood or irritability | 153 | 9.8±0.4 | 465 | 5.2±0.2 | <0.001** |
| Difficulty concentrating | 187 | 8.7±0.3 | 431 | 5.3±0.3 | <0.001** |
| Flu-like symptoms | 80 | 10.2±0.6 | 538 | 5.7±0.2 | <0.001** |
| Significant suffering | 67 | 9.8±0.6 | 551 | 5.9±0.2 | <0.001** |
| **DASS-21 Scores/Caffeine Withdrawal symptoms** |  | **YES** |  | **NO** |  |
|  | **n** | **x̄ ± SD** | **n** | **x̄ ± SD** | **p** |
| Headache | 222 | 20.9±1.0 | 396 | 13.7±0.6 | <0.001** |
| Fatique or drowsiness | 219 | 21.5±1.0 | 399 | 13.4±0.6 | <0.001** |
| Depressed mood or irritability | 153 | 26.7±1.1 | 465 | 12.9±0.5 | <0.001** |
| Difficulty concentrating | 187 | 23.3±1.0 | 431 | 13.3±0.6 | <0.001** |
| Flu-like symptoms | 80 | 26.7±1.7 | 538 | 14.7±0.5 | <0.001** |
| Significant suffering | 67 | 26.9±1.7 | 551 | 15.0±0.5 | <0.001** |

**Table S3.** DASS-21 scores of Individuals Based on the Presence/Absence of Caffeine Withdrawal Symptoms

**p<0.001

| **CUDQ Scores/Caffeine Withdrawal symptoms** | **YES** | | **NO** | |  |
| --- | --- | --- | --- | --- | --- |
|  | **n** | **x̄ ± SD** | **n** | **x̄ ± SD** | **p** |
| Headache | 222 | 19.8±0.4 | 396 | 14.4±0.2 | <0.001** |
| Fatique or drowsiness | 219 | 19.5±0.4 | 399 | 14.6±0.2 | <0.001** |
| Depressed mood or irritability | 153 | 20.9±0.5 | 465 | 14.6±0.2 | <0.001** |
| Difficulty concentrating | 187 | 20.0±0.4 | 431 | 14.7±0.2 | <0.001** |
| Flu-like symptoms | 80 | 21.2±0.8 | 538 | 15.6±0.2 | <0.001** |
| Significant suffering | 67 | 23.4±0.8 | 551 | 15.5±0.2 | <0.001** |

**Table S4.** CUDQ Scores of Individuals Based on the Presence/Absence of Caffeine Withdrawal Symptoms
